# Machine learning to support social media empowered patients in cancer care and cancer treatment decisions

**DOI:** 10.1371/journal.pone.0205855

**Published:** 2018-10-18

**Authors:** Daswin De Silva, Weranja Ranasinghe, Tharindu Bandaragoda, Achini Adikari, Nishan Mills, Lahiru Iddamalgoda, Damminda Alahakoon, Nathan Lawrentschuk, Raj Persad, Evgeny Osipov, Richard Gray, Damien Bolton

**Affiliations:** 1 Research Centre for Data Analytics and Cognition, La Trobe University, Victoria, Australia; 2 Austin Hospital, Heidelberg, Victoria, Australia; 3 North Bristol, NHS Trust, Bristol, United Kingdom; 4 Department of Computer Science, Electrical and Space Engineering, Luleå University of Technology, Luleå, Sweden; 5 School of Nursing and Midwifery, La Trobe University, Victoria, Australia; National Institutes of Health, UNITED STATES

## Abstract

**Background:**

A primary variant of social media, online support groups (OSG) extend beyond the standard definition to incorporate a dimension of advice, support and guidance for patients. OSG are complementary, yet significant adjunct to patient journeys. Machine learning and natural language processing techniques can be applied to these large volumes of unstructured text discussions accumulated in OSG for intelligent extraction of patient-reported demographics, behaviours, decisions, treatment, side effects and expressions of emotions. New insights from the fusion and synthesis of such diverse patient-reported information, as expressed throughout the patient journey from diagnosis to treatment and recovery, can contribute towards informed decision-making on personalized healthcare delivery and the development of healthcare policy guidelines.

**Methods and findings:**

We have designed and developed an artificial intelligence based analytics framework using machine learning and natural language processing techniques for intelligent analysis and automated aggregation of patient information and interaction trajectories in online support groups. Alongside the social interactions aspect, patient behaviours, decisions, demographics, clinical factors, emotions, as subsequently expressed over time, are extracted and analysed. More specifically, we utilised this platform to investigate the impact of online social influences on the intimate decision scenario of selecting a treatment type, recovery after treatment, side effects and emotions expressed over time, using prostate cancer as a model. Results manifest the three major decision-making behaviours among patients, Paternalistic group, Autonomous group and Shared group. Furthermore, each group demonstrated diverse behaviours in post-decision discussions on clinical outcomes, advice and expressions of emotion during the twelve months following treatment. Over time, the transition of patients from information and emotional support seeking behaviours to providers of information and emotional support to other patients was also observed.

**Conclusions:**

Findings from this study are a rigorous indication of the expectations of social media empowered patients, their potential for individualised decision-making, clinical and emotional needs. The increasing popularity of OSG further confirms that it is timely for clinicians to consider patient voices as expressed in OSG. We have successfully demonstrated that the proposed platform can be utilised to investigate, analyse and derive actionable insights from patient-reported information on prostate cancer, in support of patient focused healthcare delivery. The platform can be extended and applied just as effectively to any other medical condition.

## Introduction

Online support groups (OSG) are an increasingly indispensable patient-centred resource for all medical conditions and illnesses as research shows that more than 80% of Internet users seek information related to medical or personal problems via online resources and social media platforms [1–3]. The institutional void of a resilient network of support for individuals (patients and partners) in relatable circumstances is the primary reason for this prevalence [4]. Widespread technology availability, literacy, accessibility and opportunity for archival search are some of the secondary reasons [5]. OSG are anonymous comfortable virtual spaces for patients, carers and information seekers to share experiences, seek advice, express emotions and provide emotional support [6–9].

OSG discussions are organized as discussion threads, where each thread starts with a question, comment or an experience about the corresponding patient’s health concerns. Other patients on the OSG respond to these concerns, thereby creating discussion threads.

**Fig 1:**
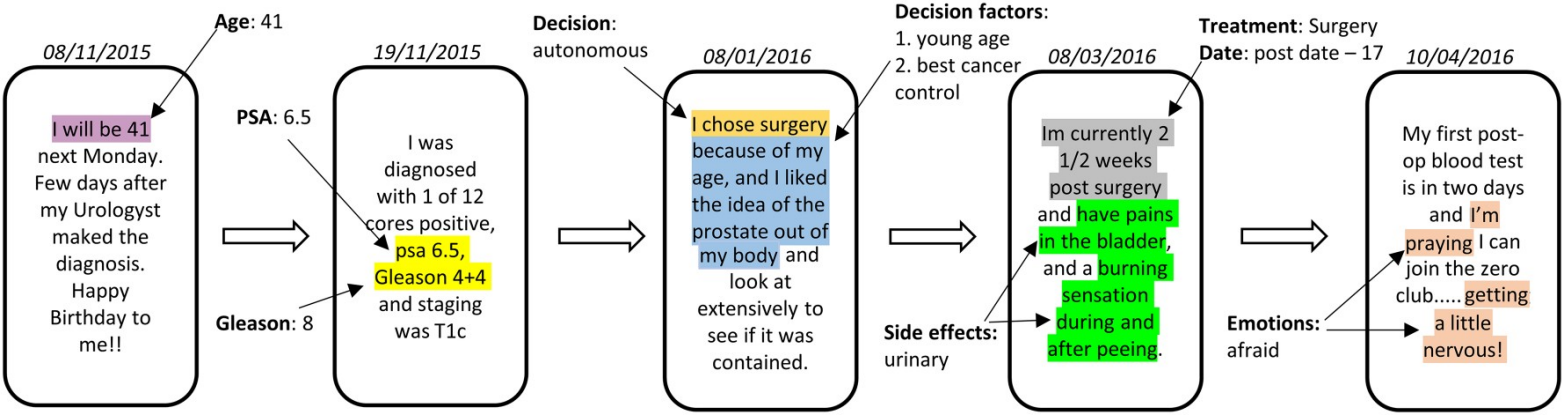
An anonymised sample (parts are omitted and rephrased to preserve privacy) of five posts by a prostate cancer patient. The highlighted excerpts are demographic, clinical, emotion expressions and decision make process related information, stated in the form of free-text.

Fig 1 presents an anonymised sample of five OSG posts by a patient, from diagnosis of cancer to four months post-surgery. It demonstrates the wealth of implicit information contained within OSG posts. Patients begin by mentioning demographic and clinical information, followed by their decision-making process, relevant decision factors and emotions, in order to seek validation from other patients [10,11]. The timeline of clinical and emotion information is implicit in the time-stamp of the post and often explicitly mentioned in the post content. However, this entire body of information is encapsulated within large volumes of unstructured text data [12] which lacks a domain-specific structure required for investigation or intervention and support by primary care providers. Advances of machine learning [13–17], deep learning [18–21] and natural language processing [22–26] present an ambitious opportunity for enabling this transition by capitalising on the prevalence of OSG and their respective networks of support.

In this paper, we present the Patient Reported Information Multidimensional Exploration (PRIME) framework for automated investigation of patient behaviours, clinical factors and patient emotions, across the temporalities of diagnosis, treatment and recovery. More specifically, we focus on the automated multi-granular extraction, analysis, classification and aggregation of decision-making behaviours, decision factors, temporality of patient interactions, temporality of clinical information and side effects, and trajectory of positive and negative emotions, in the context of decision groups, demographics and treatment type. The initial development of PRIME comprised of an ensemble of machine learning (ML) algorithms and natural language processing (NLP) techniques exclusively focused on addressing the nature, content and variety of OSG discussions [27–30]. The NLP techniques map everyday language on to ontology-driven vocabularies and thereby introduce clinical context into informal discussions. The ML algorithms distinguish between diverse patient behaviours and associate these with clinical contexts and patient demographics.

We applied PRIME on OSG for prostate cancer patients. PRIME can be seamlessly applied to any OSG focused on a different medical condition. Our selection of prostate cancer (PCa) was motivated by several factors. They are the complexity of selecting a treatment type for PCa (taking into account cancer maturity and likelihood) [31], PCa has the highest five-year relative survival rate[32], PCa is among the highest reported type of cancer[33], one of the least supported in terms of patient-centred care[34] and most in need of patient education[35].

## Related work

Numerous research endeavours have been reported in recent literature for determining patient factors from free text discussions in OSG. A majority of these are qualitative approaches, based on manual categorisation of OSG posts by domain experts. The categorisations include (i) the type of support sought/provided such as emotional/informational/medical/networking [36–38], (ii) the type of emotions expressed [39–41], and (iii) other illness specific topics discussed [38,42]. A key limitation of manual categorisation is that volume is limited to several hundred discussions.

Automated intelligent text analysis methods have been proposed for the analysis of large volumes of discussions. Such methods include unsupervised approaches such as topic capturing [43] and text clustering [44,45] to understand the topics discussed in OSG posts, as well as supervised techniques to capture different categories of OSG posts based on manually coded training datasets [46]. Standard linguistic ontologies [47] to measure emotional and psychological aspects of the OSG posts [48,49] as well as deep learning based classification methods to characterise the mental state of the author based on expression of language [50], have also been reported.

However, all related work is limited to a single aspect of online support, thereby lack the capacity to investigate, analyse and derive actionable insights, over time, from diverse patient-reported information.

## Methods

### Data collection

OSG data related to PCa was collected from ten high volume active OSG focused on PCa discussions. An active OSG is defined as having at least 100 new conversations per week. From these active OSG, conversations were automatically filtered using the specific topic *‘prostate cancer’*. The collected dataset contains 609,960 conversations from 22,233 patients, comprising a text corpus of 93,606,581 word tokens.

### Inclusion criteria

Since our interest is in patient decision-making, across different PCa treatment modalities [51], we have set our inclusions criteria as patients who have self-disclosed their chosen PCa treatment and discussed the decision-making process that led to the selection. Note, that PRIME was utilised to automatically extract this information from the collected OSG discussions. A total of 6,457 patients (29%) met these inclusion criteria and thus, selected for this study. Table 1 presents the distribution of patients who met the inclusion criteria across the ten OSG.

**Table 1:** The patient distribution (inclusion criteria met) across the ten selected OSG.

Online support groups (OSG)	URL	n (% in total)
Healingwell	www.healingwell.com/community	2520 (39.0)
Cancerforums	www.cancerforums.net	873 (13.5)
Cancer Survivors Network	csn.cancer.org/forum	810 (12.5)
Healthboards	www.healthboards.com/boards	429 (6.6)
Prostatecancerinfolink	prostatecancerinfolink.ning.com/forum	396 (6.1)
Cancercompass	www.cancercompass.com	356 (5.5)
Prostatecanceruk	community.prostatecanceruk.org	308 (4.8)
Patientinfo	patient.info/forums	299 (4.6)
Ustoo	www.inspire.com/groups/us-too-prostate-cancer	236 (3.7)
Macmillanuk	community.macmillan.org.uk	230 (3.6)

### Ethical considerations

We have obtained ethics approval for this research from the La Trobe University Human Ethics Committee. All patient-reported data used in this study are non-identifying and publicly available from the corresponding OSG. The OSG does not provide access to identifying information of patients, and we have not processed any identifying information using PRIME. We have only published aggregates of the analysed data, which cannot be reverse engineered using any means for any form of re-identification.

## Patient Reported Information Multidimensional Exploration (PRIME)

**Fig 2:**
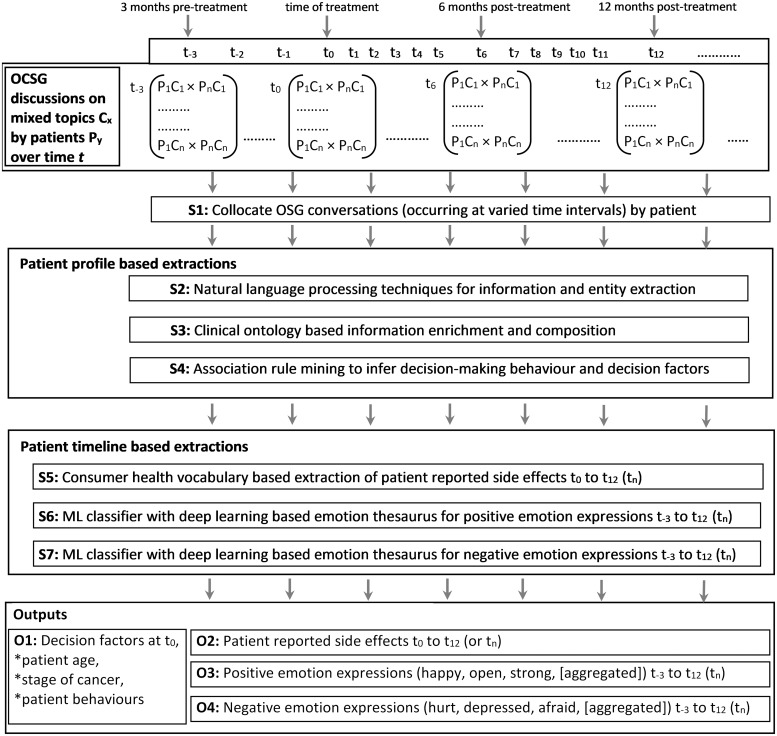
Structural and functional elements of the PRIME framework.

The PRIME framework functions in seven stages S1-S7 as depicted in Fig 2. Stages S1-S3 are based on our previous work [27–29]. All stages are delineated in the following subsections.

### Stages S1-S3

An OSG comprises a large number of discussions where patients contribute their decisions, experiences and opinions at different stages of their patient journey from diagnosis to post-treatment. The naturally occurring order of discussions provides a multitude of granular and aggregate information on patient behaviours, side effects and emotion expressions over time. However, posts by a single patient are scattered over multiple discussions. Therefore, *S1*, collocates conversations by a single patient, chronologically ordered based on timestamp. In *S2*, NLP based information retrieval techniques [24] are used to process the text corpus and subsequently, machine learning algorithms for classification are utilised to extract demographic information mentioned in free text [28]. Next, *S3* enriches this multidimensional information model with prostate cancer specific clinical information, which are important to categorise patients based on the stage of cancer. In relations to prostate cancer, Gleason and PSA information are key determinants that are extracted. In *S3*, association rules and extracts from clinical ontologies [52,53] are utilised to capture multiple narrative styles for Gleason and PSA mentions(e.g., ‘GS3+3’, ‘Gleason 7’). Subsequently, a classifier based on regular expressions was developed to capture the numerical details of Gleason and PSA scores. Further deliberations can be found in [27–29].

### Stage S4

Each patient’s decision making behaviour was inferred based on three well-established decision behaviour groups [54,55] (i) Paternalistic: those who strictly adhere to clinician recommendations, (ii) Autonomous: those who are solely driven by personal preference, and (iii) Shared: a mixed group whose decisions are based on both clinician recommendations and personal preferences. We hypothesised this information is encapsulated in the OSG posts which contain mentions of treatment options for prostate cancer.

A set of template patterns was engineered to capture sentences that describe that either individual has taken the decision (Autonomous) or the treatment option was recommended by a clinician (Paternalistic). The template patterns are as follows:

- Autonomous template: <I/We> <words>* <DECIDE> <words>* <TREATMENT>
- Paternalistic template: <DOCTOR> <words>* <RECOMMEND> <words>* <TREATMENT>

Note that <words>* denotes zero or multiple words in-between, and uppercase terms are template terms which consider a set of synonym terms (word/phrase). Table 2 shows a selected sample of terms for each template term.

**Table 2:** Sample terms for template decision making terms.

Template term	Candidate sample terms
Decide	decided, chosen, wind up going, made the call, settled, opted, went for, took the option, end up
Recommend	recommend, recommended, prescribe, prescribed, advised, advise, endorse, endorsed, advocate
Doctor	doctor, doc, surgeon, urologist, uro, specialist, consultant, radiologist, oncologist, radiotherapist
Treatment	Surgery: surgery, davinci, da vinci, robotic, prostatectomy, ralp, rrp, lrp, rpp, key hole, open op
	Radiation: radiation, imrt, brachytherapy, radiotherapy, seed therapy, brachy, seed implant, ebrt
	Surveillance: surveillance, AS, watch and wait,

Multiple decision factors, both clinical/non-clinical affect the treatment decision-making process. As shown in Fig 1, patients often mention decision factors alongside the mentions of the treatment decision. These decision factors were captured using a thesaurus of consumer health terms. Initially, a list of common decision-making factors related to prostate cancer was created based on existing literature [42,56–59] and further validated by clinicians. This list includes medical concerns such as side effects, doctor skills, and best cancer control as well as socio-demographic reasons such as age, fast recovery and financial concerns. Association rule mining was conducted on the corpus to determine decision factors for each patient from this list.

### Stage S5

Stage *S5* onwards, PRIME framework incorporates the time dimension of OSG discussions and patient interactions. A patient event timeline is automatically generated for each individual based on the self-disclosed side effects captured in *S5* and positive/negative emotions captured in *S6-S7*. Each patient timeline is time-normalised by considering the treatment month captured in *S4* as t_0_. The events (side effects and emotions) are aggregated monthly based on the reported timestamp, and the timeline is generated from three months pre-treatment (t_-3_) to 12 months post-treatment (t_12_) based on the available information. *S5* captures the self-disclosure of side effects and grouped into four key categories: *urinary*, *sexual*, *bowel* and *other* which represent side effects of prostate cancer treatments. Note that, *other* represents the miscellaneous side effects such as *hernia*, *clots* etc. A thesaurus of relevant terms (words/phrases) was used to capture any mentioned of an occurrence of side effects and map such mentions to the timeline based on the associated timestamp. Even though the clinical terms for side effects are well defined and recorded in clinical ontologies [52], individuals often describe side effects using everyday language (e.g., urinary incontinence described as leakage, leak, drip), which are not found in clinical ontologies [60]. Therefore, a sample of OSG posts was examined by a team of clinical experts, and consumer health terms related to each side effect category were captured and included in the thesaurus.

### Stages S6-S7

As established in the clinical literature, OSG are an accommodative environment for patients to freely express emotions [61,62]. Expressions of emotion reflect Quality of Life (QoL) measures such as living with the condition, the impact of treatment preferences and side effects. In S6, a machine learning technique incorporating a domain-specific vocabulary of positive emotion expressions determines explicit and implicit instances of positive emotions, emotion categories and associated strength of emotion and in S7, this was extended to negative emotion expressions.

Many psychological emotional models have been proposed in the research literature to represent human emotions. These range from the two-dimensional valence-arousal model [63] to multi-dimensional models such as emotion wheel [64]. While such models serve as the theoretical basis for emotion representation, computational implementations must capture expressions of emotion from textual discourse. For example, sentiment analysis techniques are the computational implementation of the valence-arousal model [65], which provide a signed real-value as the sentiment score, where the sign (positive/negative) represents the valence and the absolute value of score represents arousal. Although sentiment analysis techniques are relatively mature and commonly used for capturing emotions, the two dimensional model is coarse-grained for representing complex emotional states of OSG users. Therefore, we developed a a new machine learning technique based on the Emotion Wheel [64,66] to capture a multi-dimensional representation of emotions.

Emotion Wheel has eight primary emotions (joy, trust, surprise, sadness, disgust, anger, anticipation and fear) and further eight secondary emotions which are derived using combinations of primary emotions (e.g., love: joy+ trust). These 16 emotions (primary and secondary) specified in the Emotion Wheel were incorporated as the emotional dimensions in the proposed computational model. The emotional intensity of each emotion is determined based on the proportion of relevant emotional terms present in each OSG post, resulting in a 16-dimensional real-valued emotion vector for each OSG post.

**Fig 3:**
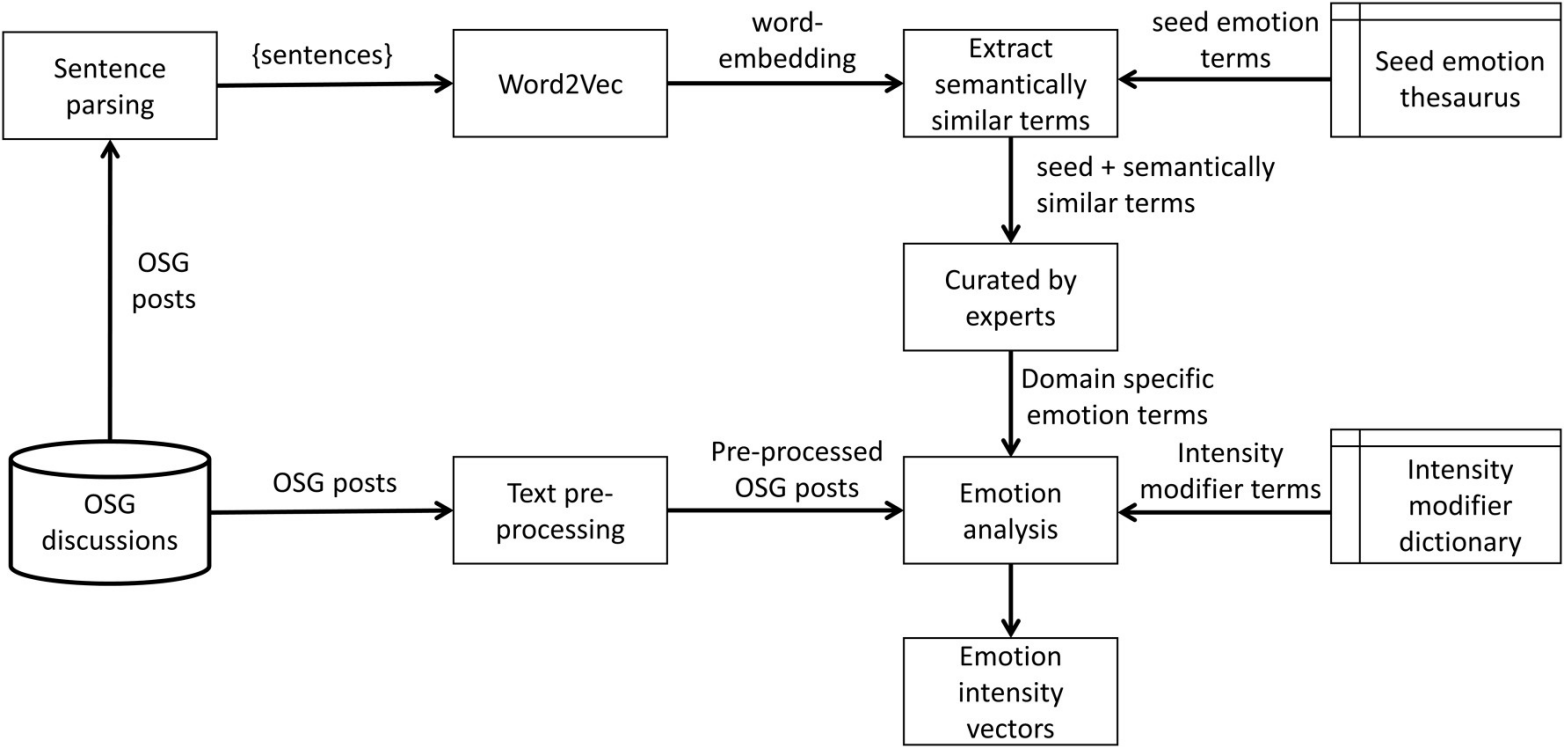
The proposed technique for emotion extraction.

Fig 3 presents the implemented technique for emotion extraction. The relevant terms for each emotion are obtained using a two-step process. First, a seed emotion term thesaurus is constructed for each emotion based on a list of feeling words used for mental status exams [67], which contains emotion terms for each of the 16 emotions.

Expanding a seed list of lexicons is a tedious activity, which is often achieved using crowdsourcing techniques such as Amazon Mechanical Turk [68]. However, recent research [69,70] reports a semi-supervised deep learning approach using word-embedding [71]. Word-embedding learns dense vector representations of words and phrases while automatically preserving the semantic relationships that exist in the text corpus by incorporating such relations into the vector space of the word-embedding. This enables the use of linear algebra to capture different semantic relationships within word-vectors in the word-embedding. The famous example in [72] shows that the vector arithmetic of word vectors ‘King -Man + Woman’ results a word vector similar to the word vector of ‘Queen’.

Developing such a word-embedding using OSG discussions enables to capture terms used by the OSG users that are semantically similar to the seed emotional terms. We have developed a word-embedding from a large text corpus which contained a total of 4,795,428 OSG posts. This corpus was pre-processed to remove URLs, convert to lower case and then separated into sentences using the Punkt sentence tokenizer [73] available in python NLTK library [74], which has shown state-of-the-art performance when compared to other sentence tokenizers with over 90% accuracy on user generated content [75]. This tokenization has resulted in 36,222,536 sentences. This text corpus was used to train a 200 dimensional word-embedding using Word2Vec technique with skip-gram model [71] and negative-sampling [76]. We utilised the python genism [77] library for this implementation. The resulting word-embedding contains 312,196 unique terms (words and phrases).

Following the trained word-embedding, top 25 most similar terms for each seed term in the emotion thesaurus was identified using a nearest neighbour search in the embedding space using Cosine similarity. These identified terms are semantically similar terms to the seed emotion terms, in which some of the terms have the same emotional sense of the seed term while some others may not. For example, the top five nearest neighbours of *sorrowful* are *sadness*, *sincerity*, *joyful*, and *deeply saddened*, in which *joyful* is semantically similar but has the opposite emotional sense. Therefore, a further empirical validation was also conducted. The third column of Table 3 presents a sample of emotional terms captured using the above technique.

**Table 3:** Emotion categories and a sample of representative terms used for each emotion.

Emotion	Emotion terms from thesaurus	Emotion terms extracted by proposed technique
**Positive emotions**		
Happy	happy, great, joyous, glad, delighted	fab, chuffed, terrific, great news, looking forward, heart warming, uplifting, upbeat
Good	good, pleased, comfortable, relaxed, content	comfy, nice, chill, chipper, ok, okay, clear headed, cool
Alive	alive, playful, energetic, spirited, animated	chatty, perky, sociable, vibrant, vivacious, witty, easy going, peppy
Love	love, attracted, warm, passionate, affectionate	romantic, cuddly, compassionate, intimate, adore, supportive, caring
Positive	positive, eager, keen, bold, brave	smart, ambitious, proactive, cynical, insistent, willing, upbeat
Open	open, understanding, accepting, satisfied, receptive	open minded, empathetic, cooperative, accommodating, approachable, forgiving, attuned, rational
Interested	interested, fascinated, inquisitive, curious, intrigued	keen, impressed, cautious, leery, eager, intuitive, savvy, thoughtful
Strong	strong, certain, dynamic, sure, tenacious	resilient, independent, adamant, fierce, self reliant, decisive, fighter, pragmatic
**Negative emotions**		
Sad	sad, tearful, grief, sorrowful, grief	heart break, teary, lonely, weepy, crying, despairing, hurtful
Afraid	afraid, fearful, terrified, panic, worry	petrified, freaking out, apprehensive, dread, obsess, fret, nervous wreck
Hurt	hurt, deprived, pained, dejected, agonised	traumatised, bruised, shattered, ached, exhausted, cramped, numb, fatigued, strained
Angry	angry, annoy, provoke, aggressive, enraged	agitated, hostile, pissed off, argumentative, aggressive, rude, paranoid, ticked off, lashing out
Depressed	depressed, disappointed, miserable, despair, powerless	despondent, distraught, suicidal, unloved, worthless, emotionally drained, snappy
Helpless	helpless, incapable, alone, vulnerable, fatigued	insecure, tired, hopeless, powerless, defeated, overwhelmed, listless, incapacitated
Confused	confused, upset, doubtful, uncertain, hesitant	unsure, perplexed, wary, leery freaked out, iffy, bummed, taken aback
Indifferent	indifferent, insensitive, dull, reserved, lifeless	grumpy, apathetic, blunt, ignorant, emotionless, callous, crass, standoffish

Intensity modifier terms are a set of terms that increase or decrease the intensity of the emotional term. For example, the term ‘very’ increases the intensity of the emotion ‘good’ when used together, whereas, the term ‘kind of’ decreases the intensity of the emotion ‘okay’ when used together. Moreover, some terms completely negate the emotions e.g., ‘not okay’ negates the emotion expressed by ‘okay’. A thesaurus of such terms are often used in rule based sentiment analysis tools such as SentiStrength [78] and VADER [79] to improve the accuracy of the sentiment score. In this work, we have used the intensity modifier term thesaurus used in VADER [79]. S1 Fig provides the algorithm for calculating emotions vector *E*_*P*_ of a given OSG post *P*.

In summary, as explicated above, PRIME functions in seven stages S1-S7 to transform OSG discussions from unstructured text discussions in the everyday language into multi-granular, multidimensional information individualised by the patient to analyse and aggregate ‘real life’ patient reported outcomes.

### Statistical analysis

The differences in variables between the groups were analysed using the Chi-Squared test (categorical) and 2-sided student’s t-test (means). The differences in side effects at selected time points were analysed using the chi-squared test, while the student’s t-test was used to compare differences between average emotion scores. P<0.05 was used for statistical significance. Analysis was performed using SAS software version 9.4.

## Results

PRIME was applied on ten high volume active OSG focused on PCa discussions, a dataset contains 609,960 conversations from 22,233 patients, comprising a text corpus of 93,606,581 word tokens. Following results are based on the inclusion criteria for this study; patients who self-disclosed their chosen PCa treatment and discussed the decision-making process that led to the selection.

**Fig 4:**
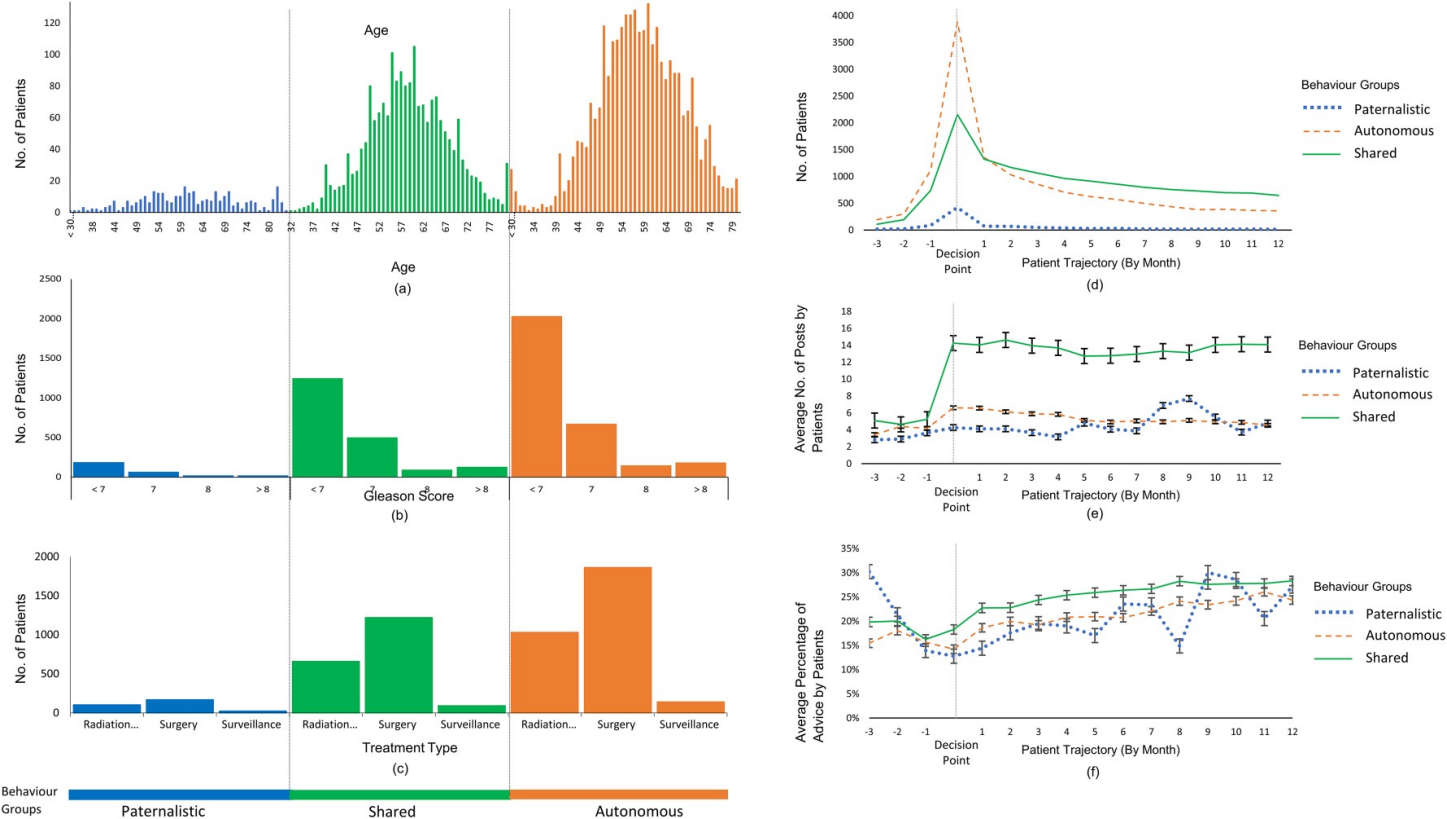
PRIME extracts multiple modalities of information individually for each patient from OSG discussions. This individual information is grouped into corresponding decision-making behaviour; Paternalistic, Autonomous and Shared groups by (a) patient age, (b) Gleason score and (c) treatment type. PRIME further generates aggregated trajectories for multiple temporal aspects of each decision-making behaviour group, before and after the decision, (d) number of patients engaged in OSG discussions, (e) average number of posts and (f) average percentage of posts containing advice by each group. The decision point is indicated by dotted vertical line in d-f.

Fig 4(a)–4(c) present the composition of each group in terms of volume, age, grading of cancer (using Gleason score) and modality of treatment. The paternalistic group is significantly smaller with a uniform distribution of age, whereas Autonomous, Shared groups are of comparable volume with approximately normal distribution of age. A high percentage of patients had Gleason<7 PCa and chose surgery as the treatment option. Fig 4(d)–4(f) represent the monthly trajectory of patient activity on OSG, three months before (-3) to 12 months following the decision. The timing of each decision is extracted by PRIME using an incremental machine learning technique [80]. In Fig 4(d), the noticeable peak of patient activity across all three groups during the period of decision-making (Paternalistic = 420, Autonomous = 3883, Shared = 2154), is an indication of active information seeking by all groups. Paternalistic and Autonomous groups reduce activity soon afterward, but the Shared group consistently participate in OSG discussions throughout the 12 months, Fig 4(e). PRIME can automatically distinguish between forum posts providing advice from those seeking answers/sharing experiences. Fig 4(f) reports the trajectory for percentage of advice posts by each group. Overall, the percentage of advice posts is lowest for the first month even though the average number of posts are highest. This number gradually increases across 12 months and interestingly, hitherto dormant Paternalistic group actively partakes in providing advice. A general trend observed in Fig 4 is that Autonomous group consistently participates over the given time period whereas Shared group demonstrates an increased interest in receiving and sharing following their treatment decision.

**Fig 5:**
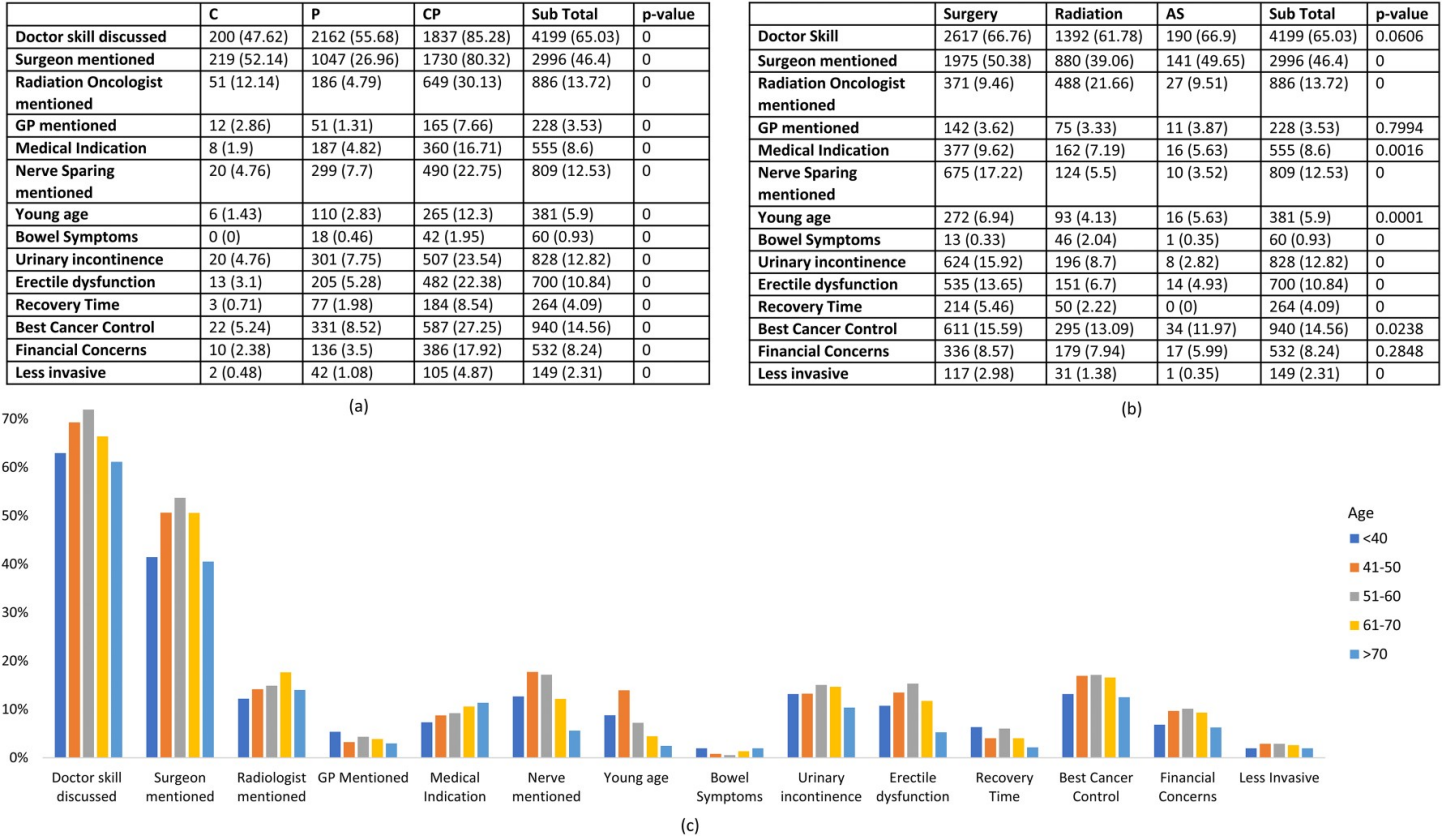
Key decision factors for PCa patients, in terms of (a) decision-making behaviour groups (b) treatment modality and (c) age groups. In (a) and (b), p-values were calculated for statistical significance.

Fig 5 illustrates the diversity of decision factors, ranging from clinical skills to financial concerns. It can be observed that ‘doctor experience’ is most influential (65%) across all three behaviour groups. Shared Group discusses all decision factors significantly more (p<0.001) than the other two groups. In treatment options, Surgery and Surveillance Groups take into consideration most factors whereas Radiation Group is more concerned about radiation oncologist (21.66%), bowel symptoms (2.04%) and financial concerns (7.94%). Age distributions (Fig 3(c)) is also diverse with fringe groups (<40 and >70 less concerned about ‘doctor experience’ than other groups and age group 51–60 is relatively more influenced by clinical factors than personal factors.

**Fig 6:**
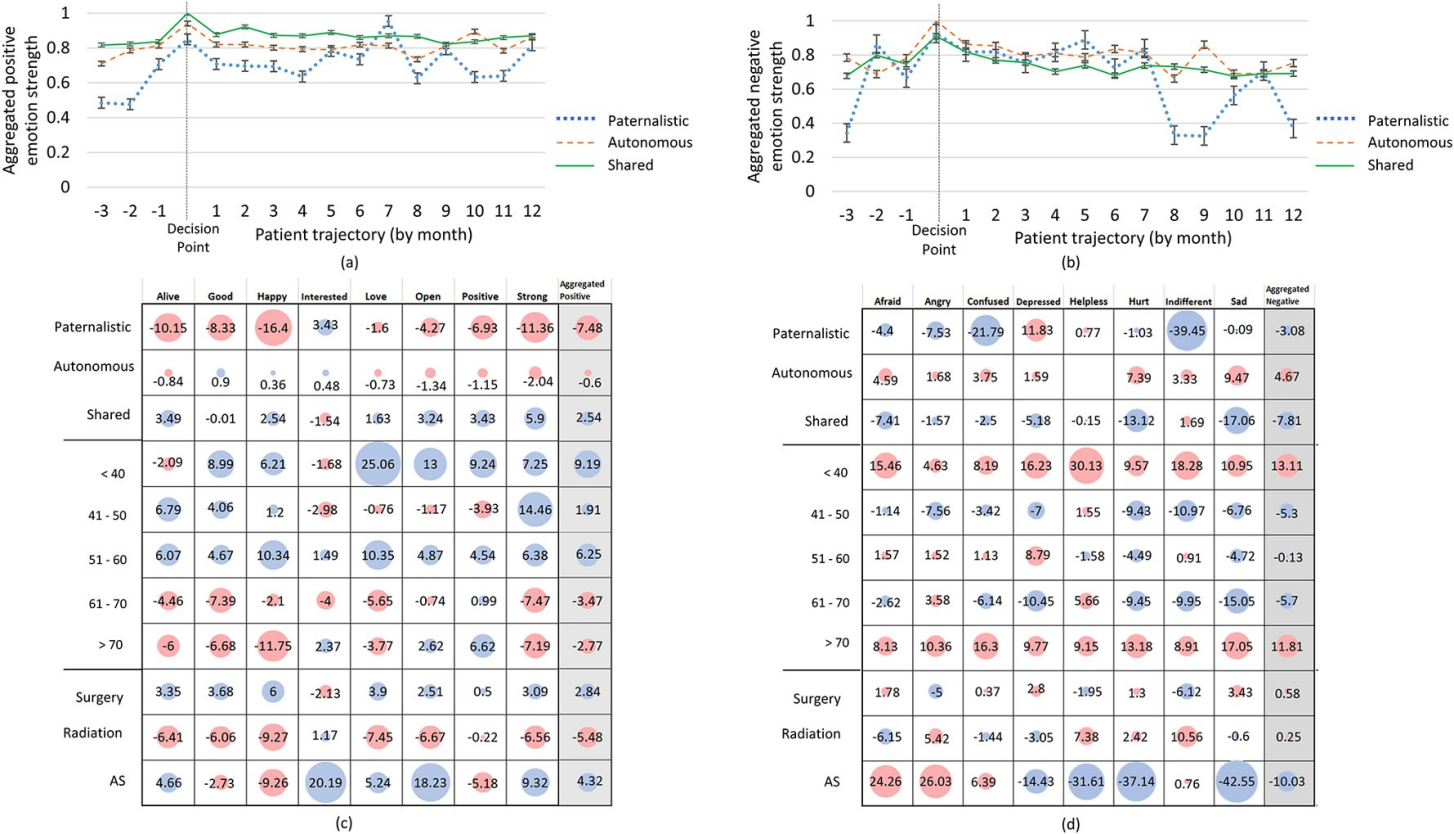
OSG provide insights into emotional journeys of patients making an intimate decision of selecting a treatment type following the diagnosis of cancer, (a) aggregated positive emotion over time, (b) aggregated negative emotion over time as well as relative strength of each distinct emotion by behaviour group, age group, treatment option, for positive emotions (c) and negative emotions (d).

Fig 6 presents a comprehensive analysis of distinct patient emotions, expressed over time—from pre-decision to recovery. Besides slight peaks at the decision point, the Shared group is mostly consistent in expressing negative and positive emotion. The paternalistic group is significantly more expressive, with far less positive emotions (aggregate of -7.48) and strongly expressed emotions related to ‘depressed’. Interestingly, this group expressed less negative emotions during 8–9 months with an immediate increase in 10–11 months. The Autonomous group demonstrate a similar pattern, less remarkably, during 8–9 months. Age group <40 consistently express above average positive emotions, with ‘love’ most expressed. On negative emotions, <40 and >70 groups are consistently above average with Surveillance group significantly below average.

**Fig 7:**
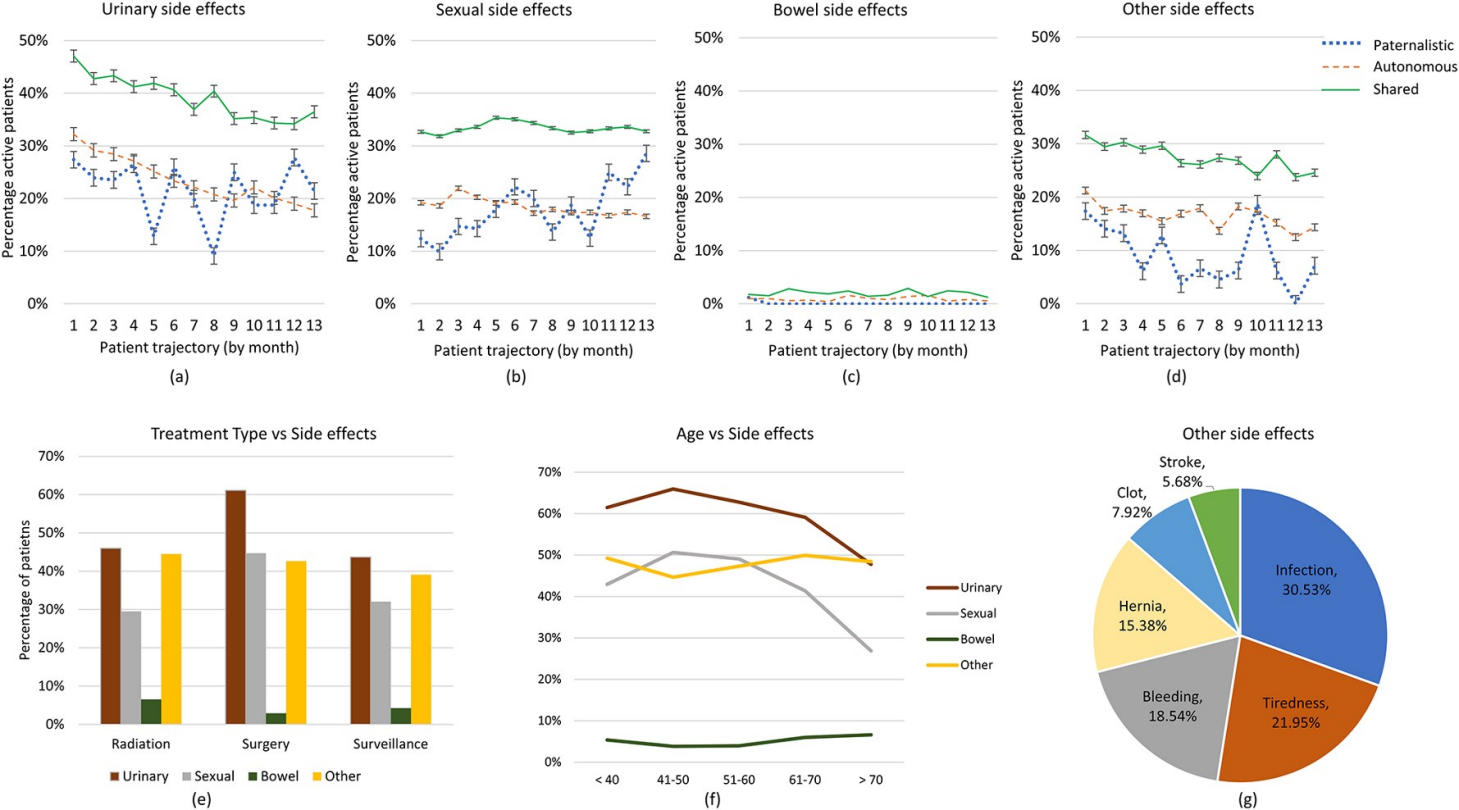
PRIME extracts and associates side effects mentioned and discussed on OSG with all other aspects of extracted patient information to generate trajectories for behaviour groups (a) urinary side effects, (b) sexual side effects, (c) bowel side effects and (d) other side effects. Further illustrated (e) side effects by treatment options, (f) side effects by age groups and (g) the general composition of other side effects.

Fig 7 reflects on side effects, with a higher percentage of Shared group reporting all side effects than the other two groups. Shared and Autonomous groups are initially affected by significant urinary side effects (Fig 7(a)) which gradual decline over time, in contrast to the Paternalistic Group which has fluctuations over time. Shared and Autonomous groups are consistently affected by sexual side effects (Fig 7(b)) while Paternalistic group shows an increase over time, reaching a level equivalent to that of the Shared group by month 12. Bowel side effects are least mentioned as the numbers opting for radiation treatment are significantly less. Fig 7(d), Paternalistic group exhibit a peak in other side effects (mainly, infections and bleeding) during the tenth month. As anticipated, sexual and urinary side effects are strongly expressed by younger age groups whereas bowel and other side effects are more consistent across all age groups, Fig 7(f).

## Discussion

Results generated by our PRIME framework strongly correlate with three major patient behaviour groups (Autonomous, Paternalistic and Shared) [54]. All groups actively sought information on OSG: The Shared group provided consistent, prolonged interactions, sharing their positive and negative emotions, experiences and advice, while the Paternalistic group were more expressive, especially with negative emotions but contributed to the OSG with advice many months post-treatment. The Autonomous group only sought advice and contributed minimally to conversations on OSG. These ecosystem-like interactions indicate the self-sufficient nature of OSG where patient voices are prominently and equally represented. Thereby, it is timely and relevant for primary care providers to accept OSG as an adjunct to cancer care and consider participating in OSG through artificial intelligence enabled optimised moderation and streamlined intervention.

In healthcare decision-making, patients continue to be an unheard, often forgotten voice [81,82]. Despite stringent efforts to advance the paradigm of patient-centred care [83,84], the importance of shared decision-making continues to be overlooked [85]. It is argued that patients should be provided necessary tools to gather information, know their decision options, scenarios and consequences for shared decision-making to be effective [86]. The significance of emotional support that allows patients to freely express values and preferences and ask questions without clinician obstruction is also highlighted [87]. The proliferation of OSG is a clear indication that patients and carers are bridging this gap by seeking (and providing) this service extraneous to healthcare providers and institutions. Further, OSG provide information, decision options and emotional support with the added advantage of a geographically dispersed community of individuals who are undergoing/have undergone similar circumstances [88,89].

Besides fulfilling the essential role of decision support for patient-centred care, OSG make a further paramount contribution as a medium for post-decision conversations on information exchange and emotional support. This is seen to be instrumental in addressing the ‘out of sight out of mind’ dilemma that arises due to periodic and/or occasional clinician consultations during the recovery phase. Patients who have undergone similar treatment are willing to share their experience, offer advice and emotional support during this crucial recovery period. Although patients with similar experiences provide each other support, OSG are peer to peer and unregulated which can be challenging for optimal healthcare. Therefore, healthcare providers must identify specific patient needs communicated on OSG, in order to optimise delivery of care and ensure that patients don’t extensively depend on their peers for healthcare advice. However, healthcare providers and institutions are progressively limited in their scope of reach and service, due to increased demand, financial constraints, resource limitations and employee turnover [90,91]. It is difficult to transition from disease-centred to patient-centred healthcare delivery in such volatile settings. With increased utilisation of OSG and the increasing presence of social media empowered patients, the medical support network for cancer care must evolve to integrate these platforms in order to provide optimal and individualised care that is clinically appropriate for patients with cancer. As explicated in this study, the PRIME framework provides significant evidence supporting the need for an optimised, cost-effective, and integrated platform for patient focused healthcare delivery.

## Conclusion

In summary, PRIME is an artificial intelligence based analytics framework for supporting social media empowered patients. It can be used for automatic aggregation and investigation of patient decision-making behaviours, decision factors, social interaction trajectory pre-/post- decision-making as well as positive and negative emotion trajectory pre-/post- decision-making. We have demonstrated these novel functionalities on patients with prostate cancer, from diagnosis to treatment and recovery. PRIME demonstrates, quantitatively, how patients in OSG evolve from being information seekers to providers, over time as they progress from treatment to recovery. Automatic aggregation and profiling patients, using machine learning and natural language processing, based on their decision-making characteristics, side effects and emotions indicate the practical value of PRIME towards informed decision-making on personalized healthcare delivery and the development of policy guidelines for primary care moderation and interventions, by clinicians, psychologists and other cancer care providers.

## Supporting information

S1 Fig Algorithm for determining the 16-dimensional emotion vector *E*_*P*_ of a given OSG post *P*. (PDF)
