# Supplementary material for: Machine learning to support social media empowered patients in cancer care and cancer treatment decisions
Source: PLoS One. 2018 Oct 18;13(10):e0205855. doi: 10.1371/journal.pone.0205855 (PMC6193663; doi:10.1371/journal.pone.0205855)
Supplement: S1 Fig — (PDF) [file pone.0205855.s001.pdf]

```
Input: P- OSG post, T_E- Emotion thesaurus, T_m- Intensity modifier
       thesaurus
Output: E(P) - Emotion vector of OSG post P
E(P) ← ∅
P ← Preprocess(P)      // remove URLs, numbers, punctuations
{w}_P ← Tokenize(P)          // tokenise the post into words

for i ← 1 to 16 do
    T_Ei ← T_E
    e_i ← 0
    w_prev ← null
    foreach w in {w}_P do
        // lookup w in emotion i term list
        if w ∈ T_Ei then
            e_i ← e_i + 1

            // lookup w_prev in intensity modifier thesaurus
            if w_prev ≠ null and w_prev ∈ T_m then
                e_i ← e_i + T_m(w_prev)
        w_prev ← w
    e_i ← e_i/|{w}_P|
    E(P) ← E(P) ∪ e_i
return E(P)
```

S1 Fig: Algorithm for determining the 16-dimensional emotion vector E_P of a given OSG post P
